# 3-Dimensional Human iPSC-Derived Cardiac Organoids for Antifibrotic Drug Screening

**DOI:** 10.1161/CIRCRESAHA.126.328797

**Published:** 2026-09-03

**Authors:** Hao Zhang, Rihua Huang, Phung N. Thai, Lu Ren, Rabindra V. Shivnaraine, Wenjuan Zhu, Yuanyuan Dai, Wenqiang Liu, Mengjie Xie, Xiu Liu, Renke Tan, Xiaochun Yang, Xu Cao, Xuekun Wu, Matthew A. Wu, Christopher Caridi, David Solow-Cordero, Joseph C. Wu

**Affiliations:** 1 Stanford Cardiovascular Institute (H.Z., R.H., L.R., W.Z., Y.D., W.L., M.X., X.L., R.T., X.Y., X.C., X.W., J.C.W.), Stanford University School of Medicine, Stanford, CA.; 2 Sarafan ChEM-H Nucleus, High-Throughput Screening and Innovative Medicines Accelerator (IMA; C.C., D.S.-C.), Stanford University School of Medicine, Stanford, CA.; 3 Department of Physiology, David Geffen School of Medicine at UCLA, Los Angeles, CA (H.Z., P.N.T.).; 4 Aerys Biosciences, Palo Alto, CA (R.V.S.).; 5 Greenstone Biosciences, Palo Alto, CA (R.V.S., M.A.W.).

**Keywords:** extracellular matrix, fibrosis, induced pluripotent stem cells, myocytes, cardiac, organoids

**Meet the First Author, see p e000771**

Cardiac fibrosis is a pathological feature of heart failure that exacerbates myocardial dysfunction. However, current therapies remain limited, largely due to the lack of translatable drug discovery platforms. To address this, we developed a platform based on human induced pluripotent stem cell–derived cardiac organoids (iPSC-COs) to screen antifibrotic compounds. Unlike 2-dimensional (2D) fibroblast cultures, which assess only fibroblast activation, 3D iPSC-COs capture crosstalk between cardiomyocytes and cardiac fibroblasts and recapitulate hallmark features of myocardial fibrosis, including ECM (extracellular matrix) deposition and contractile dysfunction.

We generated the iPSC-CO model by co-culturing iPSC-cardiomyocytes and iPSC-cardiac fibroblasts at a 4:1 ratio, allowing self-organization into 3D constructs that maintain stable contractility for at least 40 days (Figure [A]). To enable high-throughput screening, the iPSC-cardiac fibroblasts were differentiated from *ACTA2* (smooth muscle actin alpha 2)–Clover2 reporter iPSC lines,^1^ enabling real-time visualization of myofibroblast activation within iPSC-COs. On stimulation with the profibrotic cytokine TGF-β (transforming growth factor-β), Clover2 intensity was markedly increased, whereas treatment with either the TGF-β inhibitor SB431542 or the pulmonary fibrosis drug pirfenidone significantly attenuated fluorescence intensity. These results demonstrate that Clover2 serves as a reliable readout of antifibrotic drug response.

**Figure.**
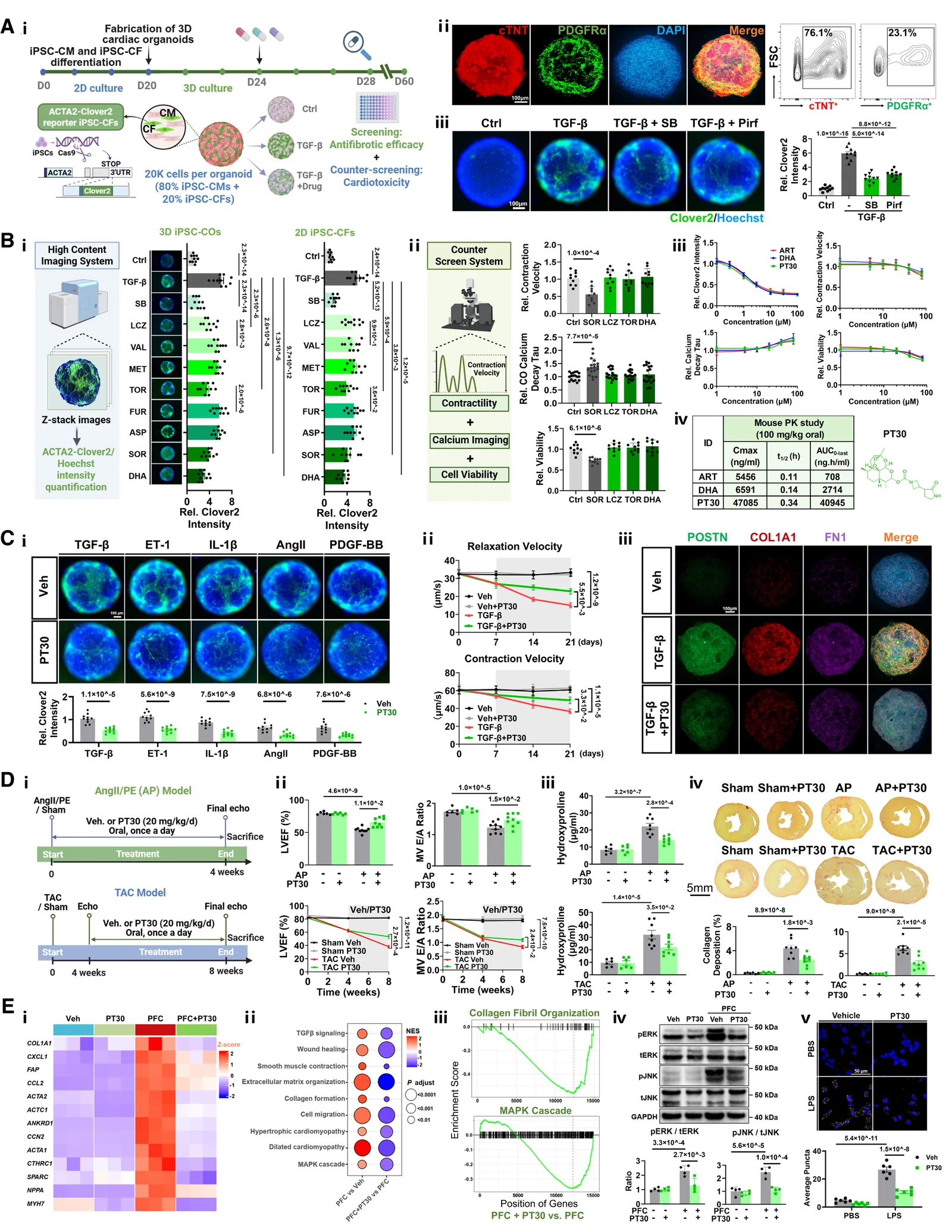
**Human induced pluripotent stem cell–derived cardiac organoid (iPSC-CO) model for identifying cardiac antifibrotic therapies. A**, **i**, A diagram of the antifibrotic drug screening platform using 3-dimensional (3D) reporter iPSC-COs. Reporter iPSC lines (from 51-year-old and 41-year-old men) were generated by inserting a Clover2 fluorescence reporter into the locus of *ACTA2* (smooth muscle actin alpha 2) using clustered regularly interspaced short palindromic repeats/clustered regularly interspaced short palindromic repeat–associated 9 as previously described.^1^ High-purity iPSC-cardiomyocytes (CMs; day 16–25) and iPSC-cardiac fibroblasts (CFs; P1–P3) were mixed at a 4:1 ratio and placed in an ultra-low attachment 96-well microplate with a cell density of 20 000 cells per well. iPSC-COs exhibit stable contractile function starting 3 days after fabrication and maintain this stability for at least 40 days. **ii**, Representative immunofluorescence images showing the expression of cTNT (CMs) and PDGFRα (CFs) in iPSC-COs, which contain a cellular center without significant necrosis. Scale bar, 100 µm. Representative flow cytometry plots showing their ratio 4 days after fabrication on the right. **iii**, Representative images and quantification showing Clover2 and Hoechst intensities in iPSC-COs treated with TGF-β (transforming growth factor-β)±SB431542 (SB; 5 μM) or pirfenidone (Pirf; 100 μg/mL) for 96 hours. The concentration of TGF-β is 5 ng/mL in all the experiments. Scale bar, 100 µm. **B**, **i**, **left**, A high-content imaging system was used to obtain Z-stacked images (20 images per cardiac organoid [CO]) with a Molecular Devices ImageXpress Micro confocal microscope. For each CO, maximum-intensity projections were generated from all acquired Z-planes. The Clover2 and Hoechst intensities were quantified from the maximum-intensity projection images. **Middle**, Representative images and quantification showing Clover2 intensities in iPSC-COs treated with TGF-β±a Food and Drug Administration–approved compound panel for 96 hours with C_max_-relevant concentrations (LCZ, LCZ696, 10 μM; VAL, valsartan, 10 μM; MET, metoprolol, 1 μM; TOR, torsemide, 5 μM; FUR, furosemide, 5 μM; ASP, aspirin, 5 μM; SOR, sorafenib, 5 μM; and DHA, dihydroartemisinin, 2 μM). SB (5 μM) was used as a positive control. **Right**, Quantification of normalized Clover2 intensity in the 2D iPSC-CFs with the same treatment, which were used as a comparison. **ii**, **left**, Schematic figure of counter-screening, including contractility measured by the Live Cell Motion Imaging System, Ca^2+^ handling measured by calcium imaging, and cell viability measured by the Promega CellTiter-Glo2.0 assay. **Right**, Quantification of normalized contraction velocity, calcium decay, and cell viability in iPSC-COs treated with different compounds. **iii**, Dose-response curves showing relative Clover2 intensity in iPSC-COs on TGF-β stimulation, treated with artesunate (ART), DHA, or PT30. Dose-response curves showing normalized contraction velocity, calcium decay, and cell viability in iPSC-COs treated with ART, DHA, or PT30. **iv**, Comparison of PT30, ART, and DHA in a mouse PK study. C_max_ represents the maximum plasma concentration. AUC represents systemic exposure. t_1/2_ represents the half-life. The structure of PT30 is shown on the right. PT30 is a new DHA derivative. **C**, **i**, Representative fluorescence images and quantification showing Clover2 intensity in iPSC-COs treated with various profibrotic stimuli, including TGF-β (5 ng/mL), ET-1 (100 ng/mL), interleukin-1β (IL-1β; 10 ng/mL), angiotensin II (AngII; 100 nmol/L), and platelet-derived growth factor-BB (PDGF-BB; 100 ng/mL) with or without PT30 for 96 hours. PT30 was used at 2 μM in all subsequent experiments. Scale bar, 100 µm. **ii**, iPSC-COs were stimulated with TGF-β for 7 days and subsequently treated with PT30 along with TGF-β for an additional 14 days. The relaxation and contraction velocities of iPSC-COs were measured at different time points. **iii**, Representative immunofluorescence images showing the expression of periostin (POSTN), COL1A1, and fibronectin (FN1) in iPSC-COs. Scale bar, 100 µm. **D**, Results of animal experiments to evaluate the antifibrotic effects of PT30, which was administered at 20 mg/kg per day orally. **i**, A schematic figure of the preventive study using a murine model of cardiac dysfunction induced by AngII/phenylephrine (PE) and of the therapeutic study, using a model induced by transverse aortic constriction (TAC) surgery. In TAC, the transverse aorta was ligated to the size of a 27-gauge needle. Ten- to 14-week-old male and female wild-type (WT) C57BL/6J mice were used in both models. **ii**, Summary data for left ventricular ejection fraction (LVEF) representing systolic function and mitral valve (MV) E/A ratio representing diastolic function. **iii**, Quantification of collagen content in the heart measured by the hydroxyproline assay. **iv**, Representative images and quantification of cardiac histological sections stained with picrosirius red to assess collagen content. Each heart was cut at the base, mid-cavity, and apex levels. Fibrosis quantification was performed in a blinded manner and expressed as the percentage of collagen area relative to the total area of the left ventricle. For the AngII/PE animal model, data were generated from n=6 (4 male mice/2 female mice) for the sham+vehicle, n=6 (4 male mice/2 female mice) for the sham+PT30, n=9 (6 male mice/3 female mice) for the AngII/PE+vehicle, and n=9 (6 male mice/3 female mice) for the AngII/PE+PT30. For the TAC animal model, data were generated from n=6 (4 male mice/2 female mice) for sham+vehicle, n=6 (4 male mice/2 female mice) for sham+PT30, n=10 (6 male mice/4 female mice) for TAC+vehicle, and n=10 (6 male mice/4 female mice) for TAC+PT30. **E**, Results of RNA sequencing in iPSC-COs treated with a profibrotic cocktail (PFC)±PT30 for 72 hours. PFC comprised TGF-β (5 ng/mL) and ET-1 (100 ng/mL). Gene set enrichment analysis (GSEA) was performed using clusterProfiler (version 4.20.0) in R. Adjusted *P* values in GSEA plots were calculated using a permutation test and then adjusted for multiple comparison testing using the Benjamini-Hochberg method. **i**, A heatmap showing representative PFC-upregulated genes reduced by PT30. **ii**, Pathway enrichment analysis showing PFC-upregulated pathways suppressed by PT30. **iii**, Representative GSEA comparing PFC+PT30 vs PFC. **iv**, Representative immunoblots and quantification showing phosphorylated and total ERK (extracellular signal-regulated kinase) and JNK in iPSC-COs treated with PFC±PT30 for 8 hours. **v**, Representative images and quantification of proximity ligation assay (PLA) fluorescence puncta (orange) showing MD2 and TLR4 interaction in the presence of LPS±PT30 in the HEK cell line with MD2 and TLR4 overexpression. Cells were pretreated with PT30 for 1 hour before exposure to LPS for 30 minutes. LPS is an MD2 ligand. Statistical analyses were performed using GraphPad Prism (version 10.1.2). Data are presented as mean±SEM. Normality was assessed using the Shapiro-Wilk test. Data were analyzed using 1-way ANOVA with Tukey multiple comparisons test (**Aiii**, **Bi**, and **Bii**), 2-way ANOVA with Tukey multiple comparisons test (**Cii**, **D**, **Eiv**, and **Ev**), and unpaired Student *t* test (**Ci**). 3′UTR indicates 3′ untranslated region; AP, angiotensin II/phenylephrine; AUC, area under the plasma concentration–time curve; COL1A1, collagen type I α1 chain; cTNT, cardiac troponin T; Ctrl, control; DAPI, 4′,6-diamidino-2-phenylindole; ET-1, endothelin-1; FSC, forward scatter; HEK, human embryonic kidney; JNK, c-Jun N-terminal kinase; LPS, lipopolysaccharide; MAPK, mitogen-activated protein kinase; MD2, myeloid differentiation factor 2; PDGFRα, platelet-derived growth factor receptor α; pERK, phosphorylated ERK; pJNK, phosphorylated JNK; PK, pharmacokinetic; tERK, total ERK; tJNK, total JNK; and TLR4, Toll-like receptor 4.

To validate the iPSC-CO platform, we conducted a pilot screen of several Food and Drug Administration–approved cardiovascular drugs, including LCZ696 (sacubitril/valsartan), valsartan, metoprolol, torsemide, furosemide, and aspirin. We also tested sorafenib, an anticancer agent with reported antifibrotic effects,^2^ as well as dihydroartemisinin, the active metabolite of the antimalarial drug artesunate, whose antifibrotic effects we previously identified.^1^ Using a high-content imaging system, we observed that LCZ696, torsemide, dihydroartemisinin, and sorafenib markedly reduced TGF-β–induced Clover2 intensity in iPSC-COs, whereas other drugs exhibited minimal effects (Figure [Bi]). These results are consistent with clinical trials showing that LCZ696 and torsemide exert greater antifibrotic effects than valsartan and furosemide, respectively.^3,4^ Notably, the potent antifibrotic effect of LCZ696 was observed only in the iPSC-CO model, but not in 2D iPSC-cardiac fibroblast cultures, reflecting the dependence of sacubitril on cardiomyocyte-derived ANP (atrial natriuretic peptide)/BNP (B-type natriuretic peptide) to attenuate myofibroblast activation.^5^

After efficacy screening, we assessed the cardiotoxicity of the hits by measuring contractile function, Ca^2+^ handling, and viability (Figure [Bii]). LCZ696, torsemide, and dihydroartemisinin showed no significant cardiotoxicity, whereas sorafenib reduced contractility, prolonged calcium transient decay, and decreased viability, indicating a limited therapeutic window consistent with its clinical cardiotoxicity. Thus, iPSC-COs enable both antifibrotic efficacy and cardiotoxicity screening.

Although artesunate and dihydroartemisinin exhibit potent antifibrotic effects, both are rapidly metabolized, limiting their systemic exposure and their potential for oral treatment of chronic fibrosis. Therefore, we leveraged this platform to test a library of new chemically modified dihydroartemisinin derivatives, designed to improve their pharmacokinetic properties. Dose-response screening in iPSC-COs demonstrated that compound PT30 retained potent antifibrotic efficacy, with a half-maximal effective concentration (EC_50_) lower than 2 μM and no significant toxicity up to 20 μM (Figure [Biii]). A subsequent oral pharmacokinetic study in mice showed that PT30 exhibited a longer half-life, higher plasma concentration, and greater systemic exposure than the parent compounds, artesunate and dihydroartemisinin (Figure [Biv]).

Next, we stimulated iPSC-COs with multiple profibrotic stimuli, and PT30 attenuated Clover2 intensity in response to all stimuli, indicating its broad-spectrum antifibrotic activity (Figure [C]). Moreover, TGF-β stimulation reduced both contraction and relaxation velocities, which resulted from increased ECM deposition in iPSC-COs, recapitulating fibrosis-induced contractile dysfunction. After TGF-β prestimulation, PT30 treatment significantly reduced ECM deposition and improved contractility, demonstrating its therapeutic effects.

To determine whether the effects observed in iPSC-COs translated in vivo, we used 2 mouse models of cardiac fibrosis (Figure [D]). In the angiotensin II/phenylephrine model, oral PT30 was administered concurrently with the angiotensin II/phenylephrine infusion to assess its preventive effects. In the transverse aortic constriction model, oral PT30 was initiated 4 weeks after surgery, when cardiac dysfunction was already established, to mimic a clinical therapeutic scenario. In both models, PT30 significantly improved cardiac function and reduced collagen deposition, consistent with the iPSC-CO observations.

To gain mechanistic insight, we performed RNA sequencing in iPSC-COs stimulated with a profibrotic cocktail (PFC), composed of TGF-β and ET-1 (endothelin-1), which induced both fibrotic and hypertrophic gene programs (Figure [Ei] through [Eiii]). Pathway enrichment analysis revealed that PT30-reversed genes were associated with fibrotic processes, including ECM organization and collagen formation, as well as cardiomyopathy pathways. These changes paralleled the reduced fibrosis and improved cardiac function in vivo. Notably, PT30 suppressed the MAPK (mitogen-activated protein kinase) cascade and markedly reduced ERK (extracellular signal–regulated kinase) and JNK (c-Jun N-terminal kinase) phosphorylation (Figure [Eiv]). To identify the upstream event, we used a proximity ligation assay, which showed that PT30 reduced the interaction between MD2 (myeloid differentiation factor 2) and TLR4 (Toll-like receptor 4) (Figure [Ev]), a molecular node we previously identified as a target of artesunate.^1^ These results indicate that PT30 suppresses MAPK activity through inhibition of the MD2-TLR4 pathway.

In summary, 3D reporter iPSC-COs provide a human-relevant platform that integrates high-throughput antifibrotic screening, cardiotoxicity counter-screening, and disease-relevant profibrotic stimulation. Before clinical translation, PT30 will require more comprehensive dose-ranging studies, pharmacokinetic optimization, and chronic toxicology studies. Broader validation across iPSC lines from diverse genetic backgrounds will further enhance the utility of the platform for predicting therapeutic responses in human cardiac fibrosis.

## Article Information

### Author Contributions

H. Zhang, R. Huang, and J.C. Wu initiated and oversaw the entire study. P.N. Thai performed the animal experiments. L. Ren, W. Liu, M. Xie, X. Yang, X. Cao, X. Wu, Y. Dai, R. Tan, and M.A. Wu performed the organoid and cell experiments. W. Zhu and X. Liu performed the bioinformatics analysis. C. Caridi and D. Solow-Cordero assisted with drug screening. H. Zhang, R. Huang, P.N. Thai, R.V. Shivnaraine, and J.C. Wu wrote and edited the manuscript.

### Data Availability

All sequencing data have been deposited in GEO under accession number GSE341748. The data, analytic methods, and study materials supporting the findings of this study are available from the corresponding authors upon reasonable request. See Major Resources Table in the Supplemental Material for article data.

### Disclosures

J.C. Wu is a cofounder and scientific advisory board member of Greenstone Biosciences and Aerys Biosciences. R.V. Shivnaraine is an employee of Greenstone Biosciences and Aerys Biosciences. The other authors report no conflicts. PT30 was provided by Greenstone Biosciences. Greenstone Biosciences has filed patents on PT30.

### Supplemental Material

Major Resources Table

## Supplementary Material
