# Supplementary material for: 3-Dimensional Human iPSC-Derived Cardiac Organoids for Antifibrotic Drug Screening
Source: Circ Res. 2026 Sep 3;139(9):e328797. doi: 10.1161/CIRCRESAHA.126.328797 (PMC13649265; doi:10.1161/CIRCRESAHA.126.328797)
Supplement: Supplementary file 1 [file res-139-e328797-s001.pdf]

Major Resources Table

In order to allow validation and replication of experiments, all essential research materials listed in the Methods should be included in the Major Resources Table below. Authors are encouraged to use public repositories for protocols, data, code, and other materials and provide persistent identifiers and/or links to repositories when available. Authors may add or delete rows as needed.

Animals (in vivo studies)

Species	Vendor or Source	Background Strain	Sex	Persistent ID / URL
Mice	Jackson lab	C57BL/6J	M/F	RRID:IMSR_JAX:000664

The laboratory animal care program at Stanford University is accredited by the Association for the Assessment and Accreditation of Laboratory Animal Care International (AAALAC International). The APLAC approval number for this study is 33803. All of the experiments were conducted in accordance with NIH and Stanford University policies. All animals were housed in individually ventilated cages (5 animals per cage) located in the same room in the animal facility managed by the Veterinary Service Center of Stanford University School of Medicine, under standard housing conditions (ambient temperature ~20-24 °C, ~50% humidity, 12 h-12 h light-dark cycles).

ARRIVE Guideline:

Blinding	Histological fibrosis quantification (picrosirius red staining) and echocardiographic measurements were performed by investigators blinded to group allocation.
Inclusion/Exclusion Criteria	Mice were included if they survived the surgical procedures (TAC) without major complications. Animals that died prematurely or experienced severe surgical complications were excluded from analysis. All remaining animals were randomized into treatment groups.
Sample Size/Power Calculation	Sample sizes for each experimental group were determined based on our laboratory's prior experience with these well-established AngII/PE and TAC models of pressure-overload-induced cardiac dysfunction, in which vehicle-treated animals reliably develop a substantial and reproducible decline in LVEF relative to sham controls. Using estimated effect sizes and variability derived from this experience, a power analysis (two-sided t-test, $\alpha = 0.05$, power $(1-\beta) = 0.80$) indicated that a smaller cohort (n = 6 per group) was sufficient for sham groups, given the minimal expected variability in the absence of disease induction, while a larger cohort (n = 9-10 per group) was used for the AngII/PE and TAC-treated groups to reliably detect the anticipated treatment effect on LVEF, consistent with the group sizes used in the present study.

Genetically Modified Animals

	Species	Vendor or Source	Background Strain	Other Information	Persistent ID / URL
**Parent - Male**					
**Parent - Female**					

Antibodies

Target antigen	Vendor or Source	Catalog #	Working concentration	Lot # (preferred but not required)	Persistent ID / URL
Cardiac Troponin T	Abcam	ab45932	1:200		https://www.abcam.com/en-us/products/primary-antibodies/cardiac-troponin-t-antibody-ab45932

DOI [to be added]

PDGFRα (CD140α)	BioLegend	323506	1:200		https://www.biolegend.com/en-us/products/pe-anti-human-cd140a-pdgfralpha-antibody-3727
Collagen 1A1	Novus Biologicals	NBP1-30054	1:200		https://www.novusbio.com/products/collagen-i-alpha-1-antibody_nbp1-30054
Periostin	Proteintech	66491-1-Ig	1:200		https://www.ptglab.com/products/Periostin-Antibody-66491-1-Ig.htm
Fibronectin	Novus Biologicals	AF1918	1:200		https://www.novusbio.com/products/fibronectin-antibody_af1918
MD2	Novus Biologicals	NBP1-75513	1:200		https://www.novusbio.com/products/md-2-antibody-1a2e3_nbp1-75513
TLR4	Proteintech	19811-1-AP	1:200		https://www.ptglab.com/products/TLR4-Antibody-19811-1-AP.htm
Phosphorylated ERK	Cell signaling	9101	1:1000		https://www.cellsignal.com/products/primary-antibodies/phospho-p44-42-mapk-erk1-2-thr202-tyr204-antibody/9101
Total ERK	Santa Cruz Biotechnology	sc-514302	1:1000		https://www.scbt.com/p/erk-1-2-antibody-c-9
Phosphorylated JNK	Cell signaling	9251	1:1000		https://www.cellsignal.com/products/primary-antibodies/phospho-sapk-jnk-thr183-tyr185-antibody/9251
Total JNK	Cell signaling	9252	1:1000		https://www.cellsignal.com/products/primary-antibodies/sapk-jnk-antibody/9252
GAPDH	Thermo Fisher	MA5-15738-HRP	1:3000		https://www.thermofisher.com/antibody/product/GAPDH-Loading-Control-Antibody-clone-GA1R-Monoclonal/MA5-15738-HRP
Alexa Fluor 488 Donkey Anti-Mouse IgG (H+L)	Thermo Fisher	A-21202	1:500		https://www.thermofisher.com/antibody/product/Donkey-anti-Mouse-IgG-H-L-Highly-Cross-Adsorbed-Secondary-Antibody-Polyclonal/A-21202
Alexa Fluor 594 Donkey Anti-Rabbit IgG (H+L)	Thermo Fisher	A-21207	1:500		https://www.thermofisher.com/antibody/product/Donkey-anti-Rabbit-IgG-H-L-Highly-Cross-Adsorbed-Secondary-Antibody-Polyclonal/A-21207
Alexa Fluor 647 Donkey Anti-Sheep IgG (H+L)	Thermo Fisher	A-21448	1:500		https://www.thermofisher.com/antibody/product/Donkey-anti-Sheep-IgG-H-L-Cross-Adsorbed-Secondary-Antibody-Polyclonal/A-21448
Anti-rabbit IgG, HRP-linked Antibody	Cell signaling	7074	1:3000		https://www.cellsignal.com/products/secondary-antibodies/anti-rabbit-igg-hrp-linked-antibody/7074
Anti-mouse IgG, HRP-linked Antibody	Cell signaling	7076	1:3000		https://www.cellsignal.com/products/secondary-antibodies/anti-mouse-igg-hrp-linked-antibody/7076

DNA/cDNA Clones

Clone Name	Sequence	Source / Repository	Persistent ID / URL

DOI [to be added]

Cultured Cells

Name	Vendor or Source	Sex (F, M, or unknown)	Persistent ID / URL
iPSC line 1	Stanford Cardiovascular Institute (SCVI) Biobank	M	SCVI113
iPSC line 2	SCVI Biobank	M	SCVI114
HEK MD2 TLR4 overexpression line	InvivoGen	unknown	293-htlr4md2cd14

Data & Code Availability

Description	Source / Repository	Persistent ID / URL
RNA seq data	GEO	GSE341748

Other

Description	Source / Repository	Persistent ID / URL

DOI [to be added]
