# Supplementary material for: 3-Dimensional Human iPSC-Derived Cardiac Organoids for Antifibrotic Drug Screening
Source: Circ Res. 2026 Sep 3;139(9):e328797. doi: 10.1161/CIRCRESAHA.126.328797 (PMC13649265; doi:10.1161/CIRCRESAHA.126.328797)

Supplementary

All the full unedited gels are for Figure Eiv

Gel 1: SCVI-113

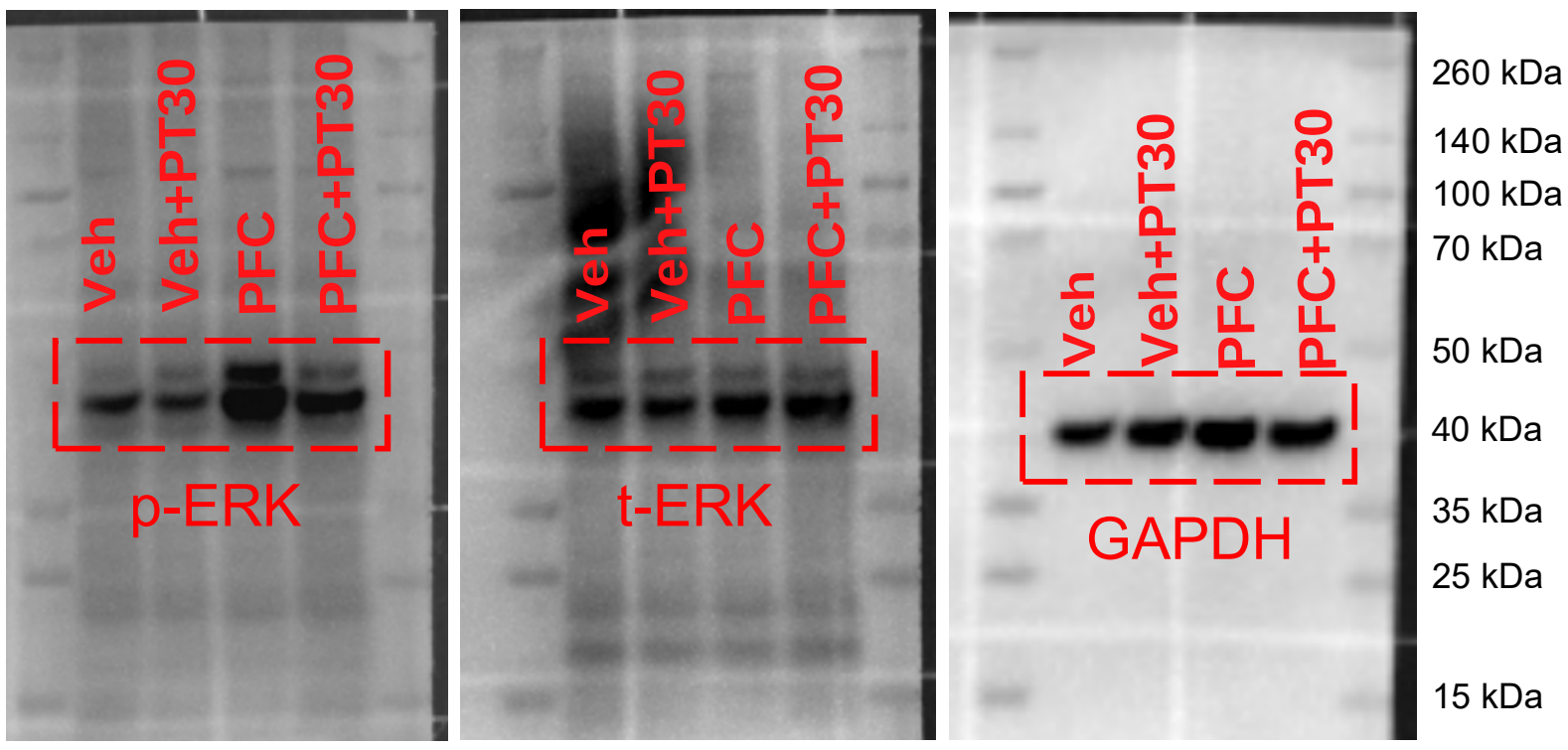

Gel 5: SCVI-113

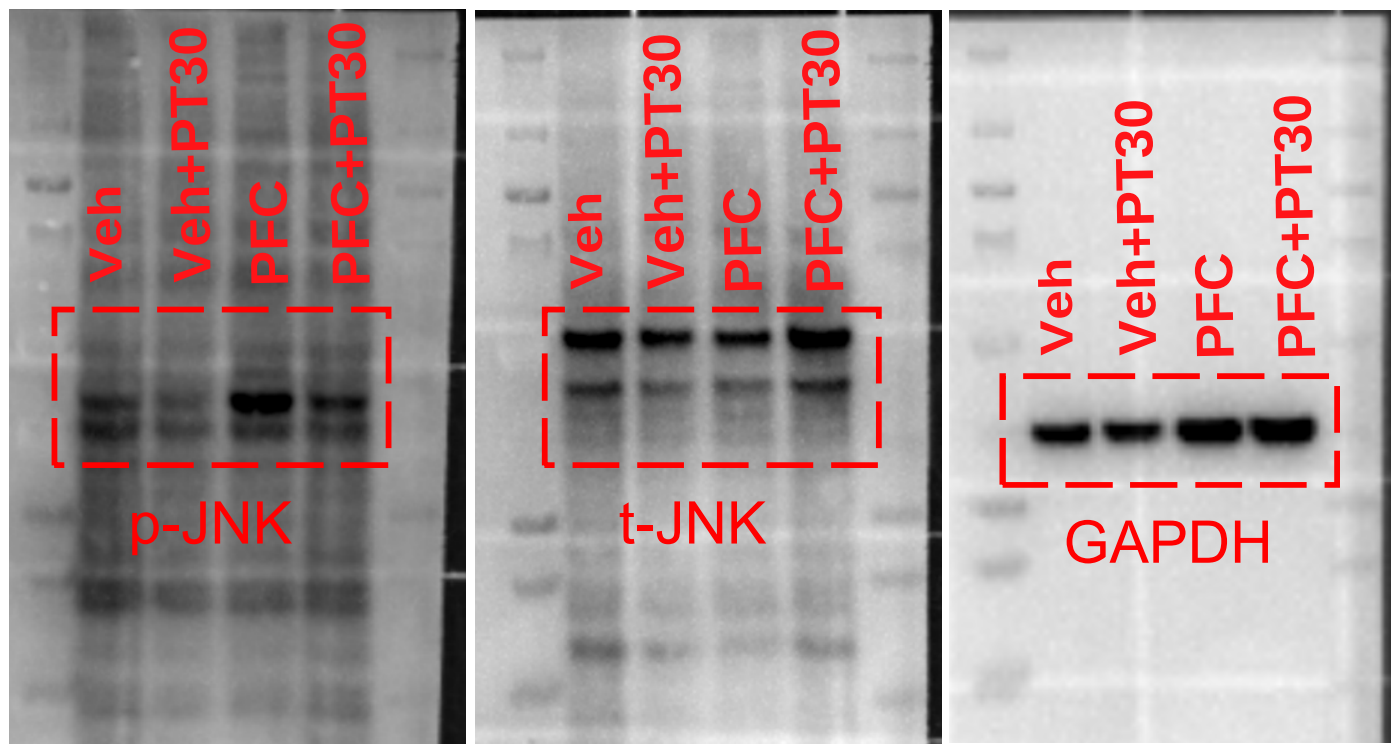

Gel 2: SCVI-113

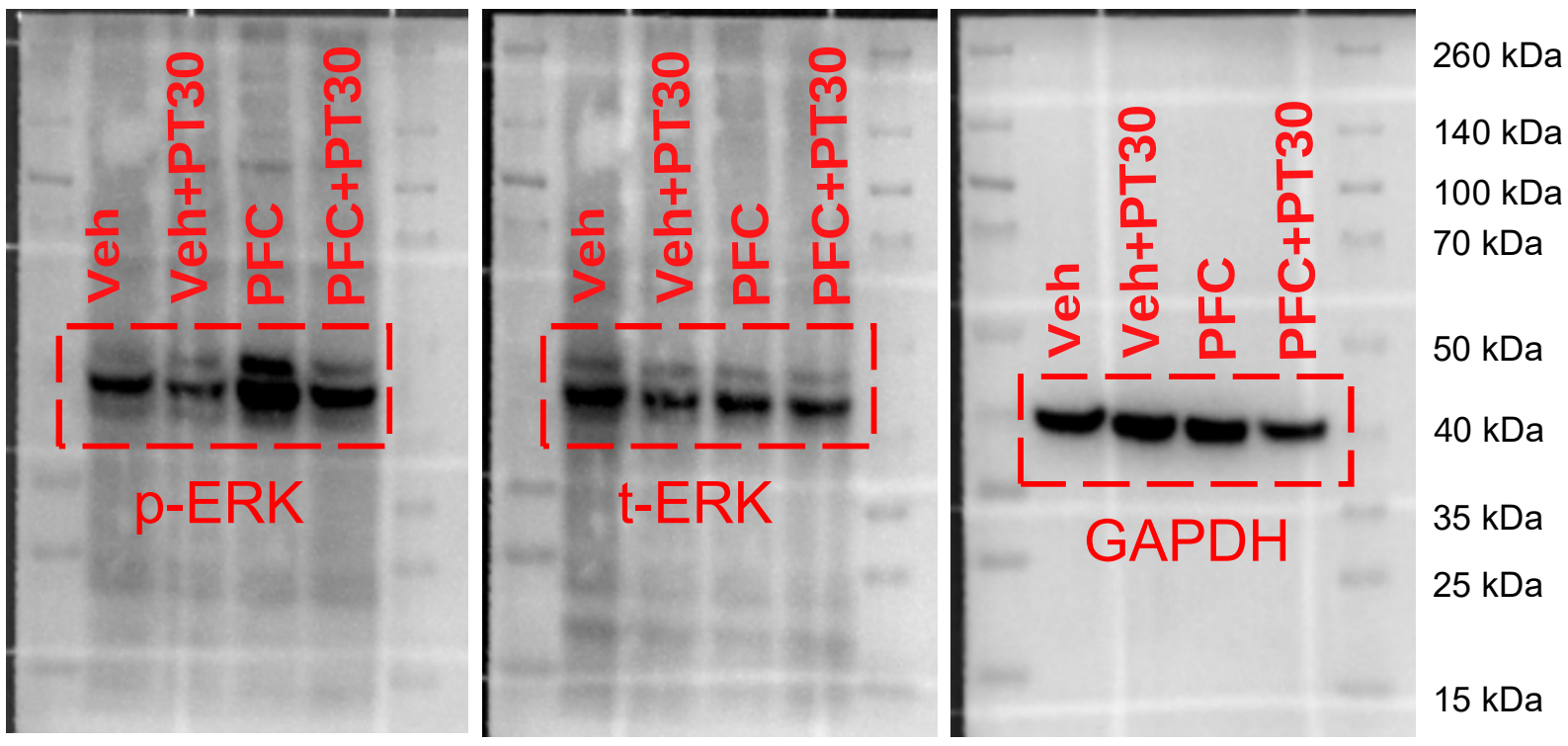

Gel 6: SCVI-113

Related to the cropped images within the manuscript

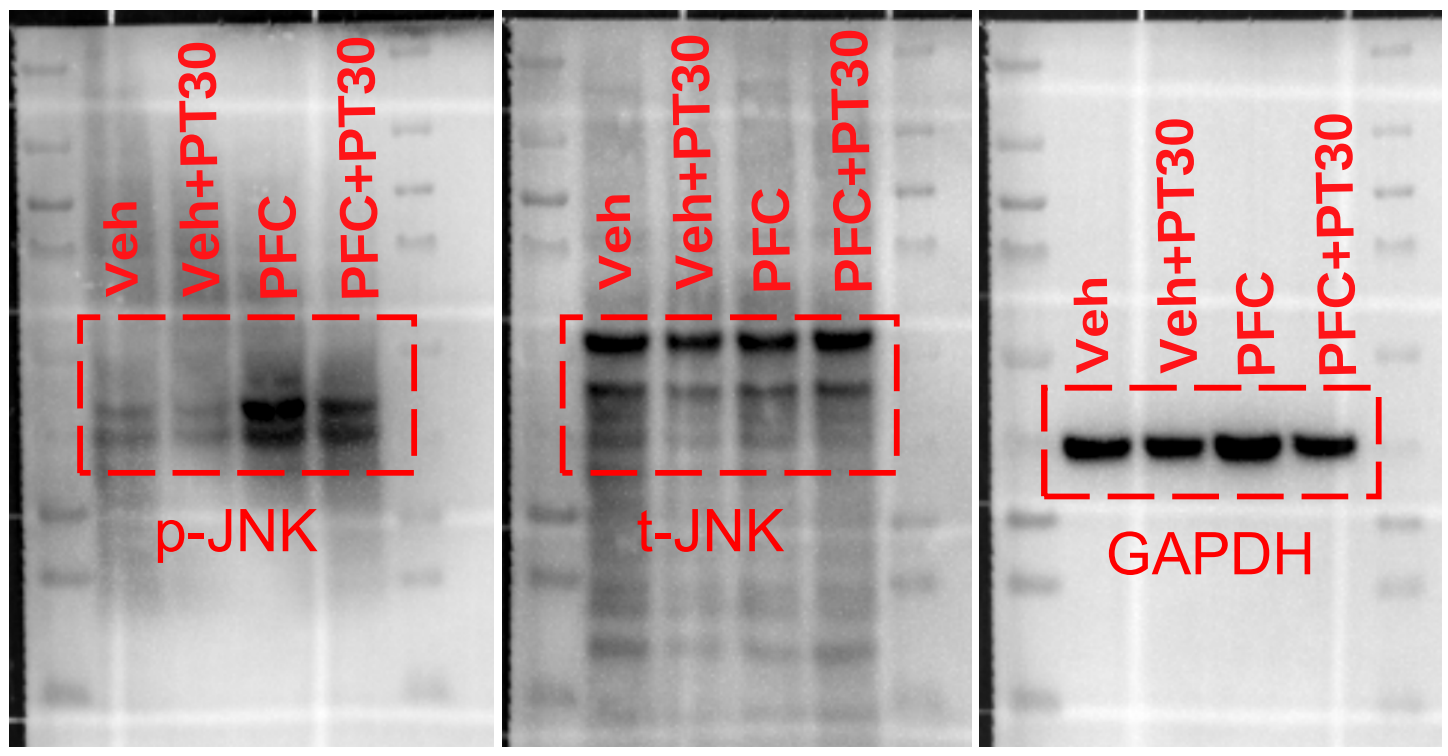

Gel 3: SCVI-114

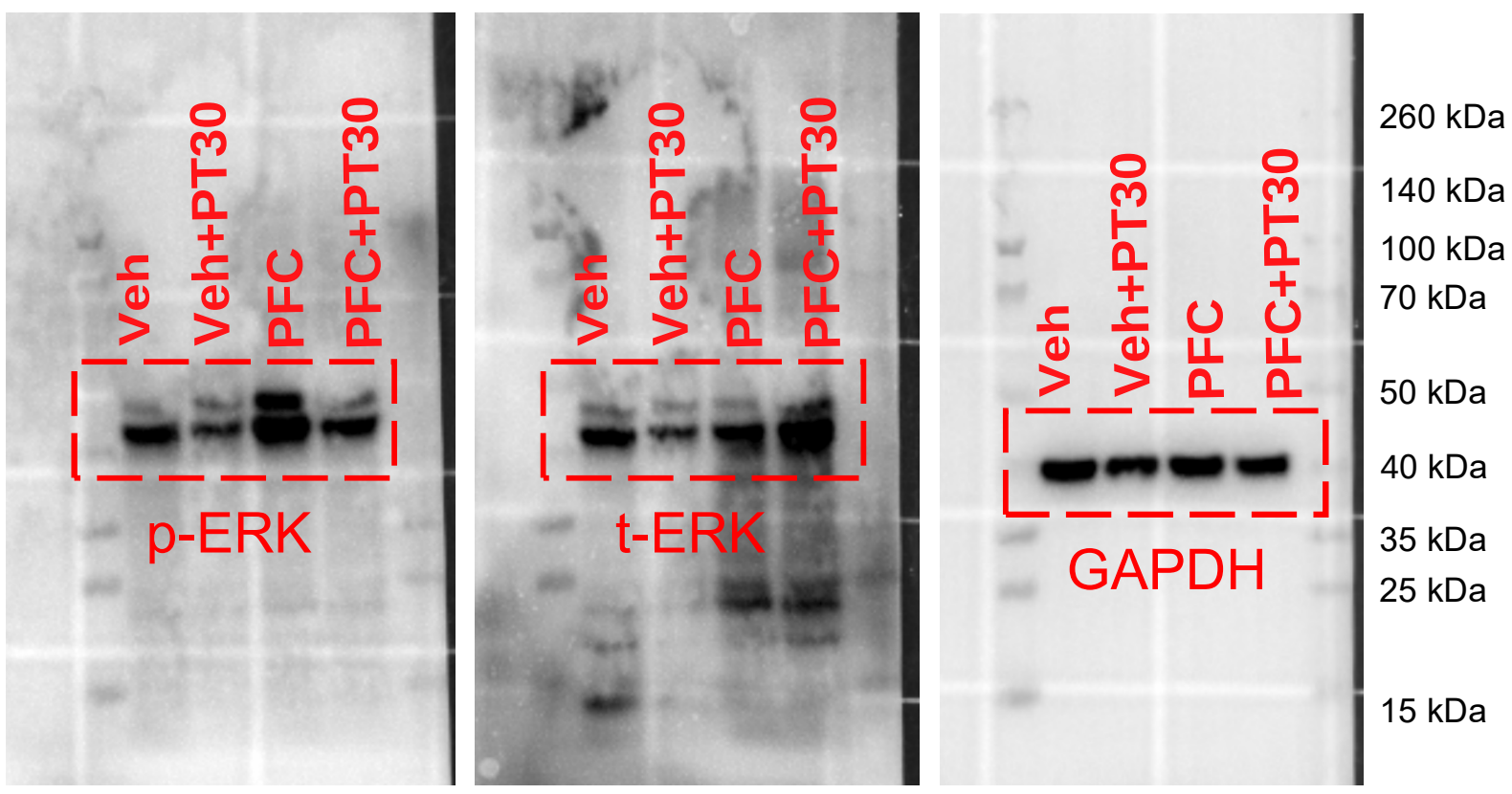

Gel 7: SCVI-114

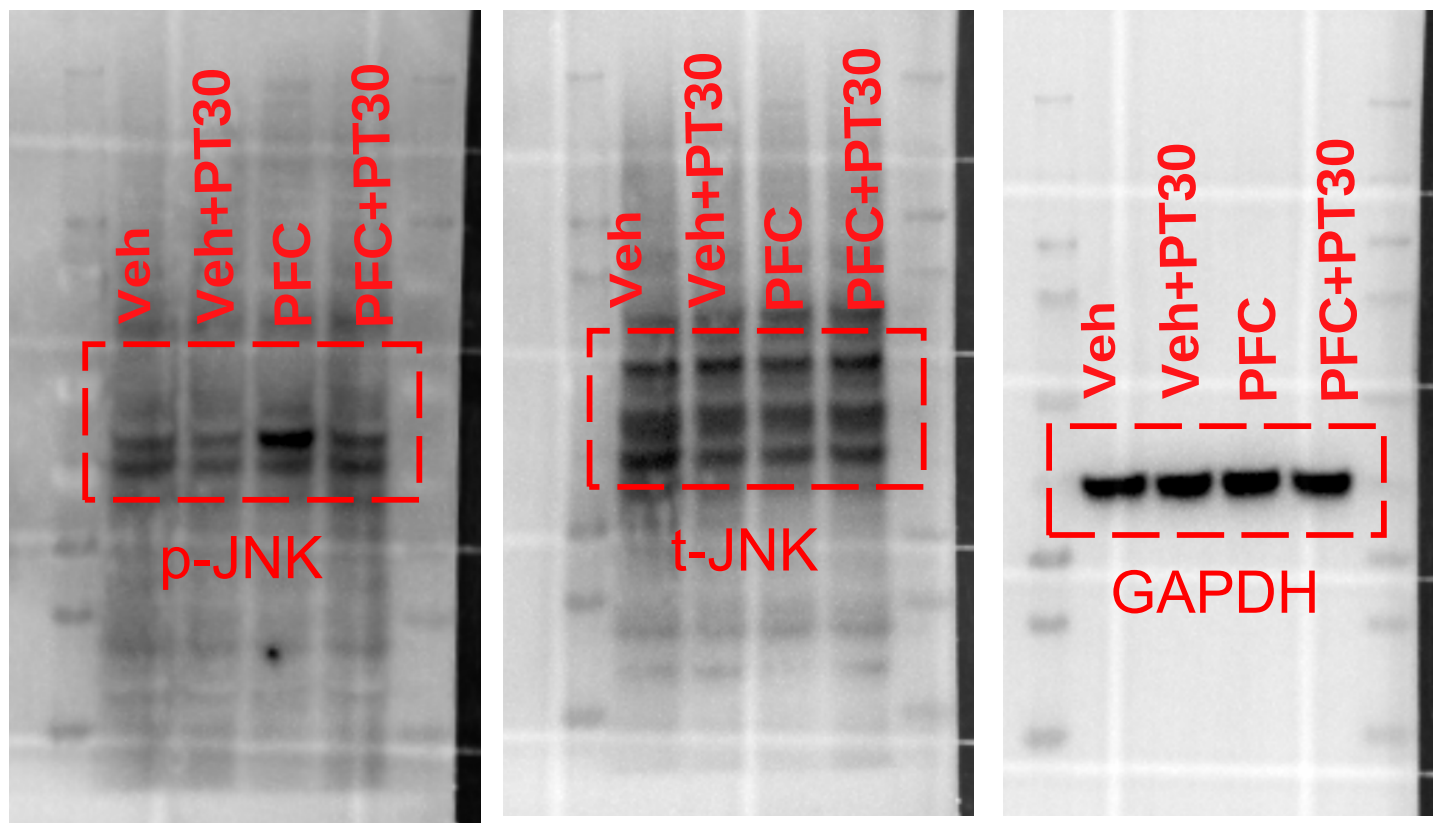

Gel 4: SCVI-114

Related to the cropped images within the manuscript

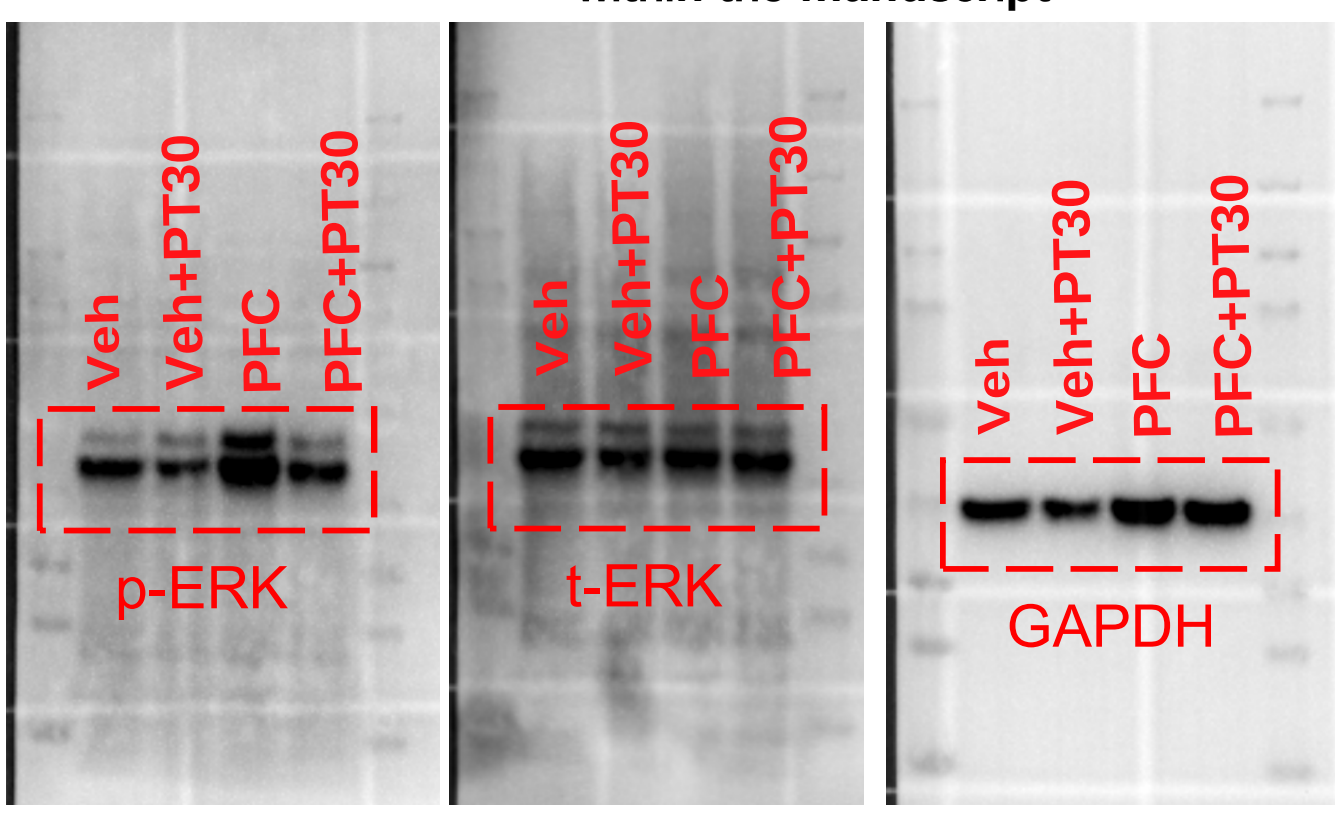

Gel 8: SCVI-114

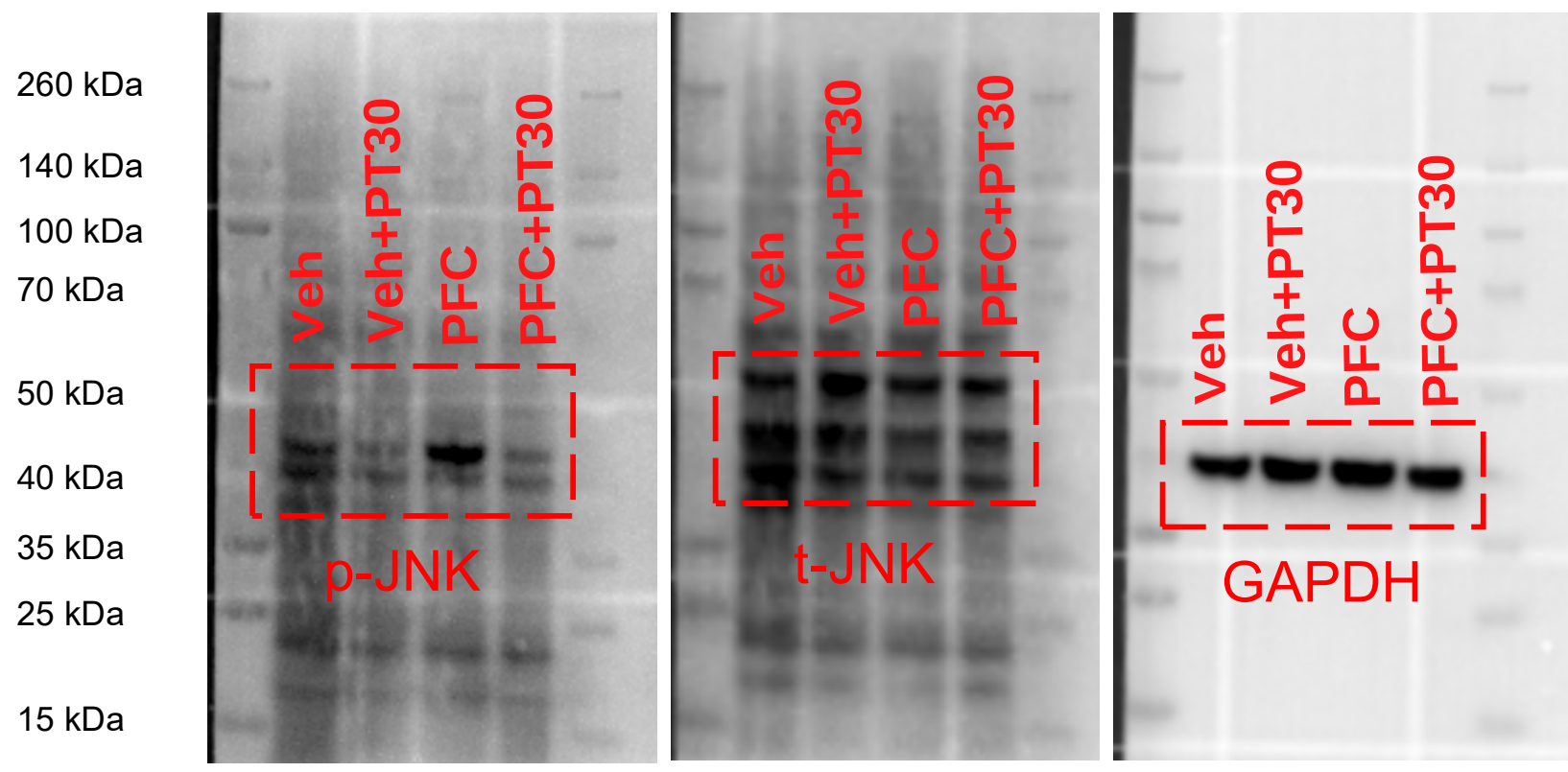

Supplement: Supplementary file 2 [file res-139-e328797-s002.pdf]
